# A decade of change in breastfeeding in China's far north-west

**DOI:** 10.1186/1746-4358-1-22

**Published:** 2006-11-24

**Authors:** Fenglian Xu, Xiaoxian Liu, Colin W Binns, Cuiqin Xiao, Jing Wu, Andy H Lee

**Affiliations:** 1 Medical College of Shihezi University; Xinjiang, 832002, PR China; 2 Tongji Medical College, Huazhong University of Science and Technology, Wuhan PRC 43001, PR China; 3 School of Public Health, Curtin University of Technology, Western Australia, 6845, Australia; 4 Shihezi Maternal and Child Health Care Institute, Xinjiang, 830000, PR China; 5 Shihezi People's Hospital, Xinjiang, 832000, PR China

## Abstract

**Background:**

There have been considerable changes in breastfeeding practices in China over the past forty years. However China is a very large country, and breastfeeding rates in different parts of China vary considerably. The objective of this paper is to identify and compare breastfeeding types and rates between 1994–1996 and 2003–2004 in Shihezi, Xinjiang Uygur Autonomous Region, PR China.

**Methods:**

In 1994–1996, a study of breastfeeding (n = 2197) was undertaken in Shihezi, Xinjiang, PR China. A decade later in 2003–2004, a longitudinal study (n = 545) of infant feeding practices was undertaken in the same area.

**Results:**

The 'any breastfeeding' rates at 1, 4 and 6 months were 94%, 82% and 78% respectively in the early 1990s. A decade later, breastfeeding at 1 month was lower, but rates at 4 and 6 months remained the same. In 2004 the 'full breastfeeding' rate at one month was significantly higher (57%) than a decade earlier (38%), but after 3 months there was a rapid decline. This reflected a shift in the way complementary foods are introduced: the initial introduction was later, but by a higher proportion of mothers.

**Conclusion:**

The rate of breastfeeding at one month is significantly lower in 2003–2004 when compared to 1994–1996. The 'full breastfeeding' rates were initially higher, but after 3 months were then lower. The Chinese national breastfeeding targets were not reached in either period of the study. These studies show the need to further promote full or exclusive breastfeeding and further longitudinal studies are necessary to provide the detailed knowledge about risk factors required for health promotion programs.

## Background

Breastfeeding is the normal way to feed all infants; immediate and long-term health problems are increased in infants fed infant formula or other foods [1]. The World Health Organization Expert Consultation recommended 'exclusive breastfeeding' for the first six months of life and continued breastfeeding up to two years of age or beyond [2]. The Chinese government set a target to achieve a national 'exclusive breastfeeding' rate at four months of 80% by 2000 [3].

There have been considerable changes in breastfeeding practices in China over the past forty years. In Beijing, 'ever breastfed' rates were reported as being 81% in urban areas and 95% in nearby rural areas [4] in the early 1950s. Then the breastfeeding rate declined significantly after the 1970s when the use of breastmilk substitutes became widespread [5]. By 1990, the breastfeeding rate still remained at a low level and a survey in Beijing City showed that breastfeeding rates were 24.7% in the first week of life and declined to 13.6% at four months [6]. Despite the fact that breastfeeding education programs commenced in 1983 in Beijing, breastfeeding rates remained at a low level for another decade. The 'any breastfeeding' rates at four months remained in the range from 13.6% to 22.0%. The Chinese government supported the Baby Friendly Hospital Initiative when it was introduced and it became policy in many hospitals in the early 1990s, and this resulted in breastfeeding rates beginning to increase again [7,8]. In a prospective study of 'rooming-in' as a breastfeeding intervention, the 'any breastfeeding' rate increased from 30.1% to 60.9% in the first week and from 11.9 to 25.8% at four months [6]. The trends of breastfeeding in other cities were similar to the changes occurring in Beijing [9].

In summary, breastfeeding rates throughout China fell during the 1970s, reaching their lowest point in the 1980s and then began to rise again in the 1990s. The rates in urban areas have generally remained lower than in rural areas [10]. However China is a very large country, geographically, as well as in population size and ethnic diversity, and breastfeeding rates in different parts of China vary considerably [11].

**Figure 1:**
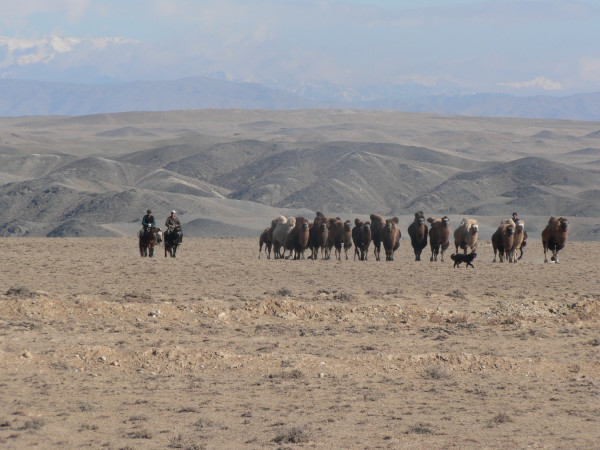
Khazak herdsmen, Xinjiang Uygur Autonomous Region.

The Xinjiang Uygur Autonomous Region (Xinjiang AR) in North-western China borders eight countries: Russia, Kazakhstan, Kirghizstan, Tajikistan, Pakistan, Mongolia, India and Afghanistan. There are more than 13 ethnic groups living in this area. Among them, the Uygur people account for 46%, Han 40% and Kazakh 7%. The population of Xinjiang had reached 19.6 million by the end of 2004. The average birth rate was 16 per thousand and death rate 5.1 per thousand [12]. Shihezi is a medium-sized city of approximately 200000 population situated in the west of the province, 2600 km from Beijing. In the past 50 years it has grown from a handful of families living around an oasis in the Gobi Desert (Figure 1) to a modern town that has its own university and medical school. The population of Shihezi is predominantly Han ethnic Chinese, while the surrounding regional areas contain a number of minority groups [12].

There have been few reported studies of infant feeding or breastfeeding rates in Shihezi or Xinjiang Province. A study from rural areas near to Shihezi showed that 'exclusive breastfeeding' in the first week of life was only 41% [13]. These rates are well below Chinese and international targets. The differences in breastfeeding patterns between the different ethnic groups in the province have recently been described [14]. The objective of this paper is to document changes in the breastfeeding rates during the decade between 1994–1996 and 2003–2004 in Shihezi, PR China.

## Methods

During the period 1994–1996, a study was undertaken in Shihezi, Xinjiang, PR China. A total of 2233 mothers of children born between June 1994 and June 1996 in Shihezi were invited to complete a questionnaire when they visited community clinics for regular physical examination or immunizations during the period July to December in 1996. Consent was obtained from 2197 parents (urban 1264, rural 933). The mothers were asked about their feeding practices on the day of interview. Data from 822 infants aged six months or younger are included in this report. The questionnaire sought information on demographic variables and infant feeding practices.

In 2003–2004, a cohort study (n = 545) of infant feeding practices was undertaken in the same area. Mothers who gave birth during 2003 and 2004 were invited to participate in the study while still in hospital and after discharge were contacted in person or by telephone at approximately monthly intervals (at 0.5, 1.5, 2.5, 3.5, 4.5 and 6 months respectively) to obtain details of infant feeding practices. In this paper, only those results of the questionnaires when the infant was one month or older are reported. The sampling sites were similar in both studies. In both cases mothers were asked about present feeding practices making comparison of the results from two studies possible.

The study in 1994–1996 was approved by the Shihezi Science and Technology Committee. Consent from parents was obtained before completing the questionnaires. The study in 2003–2003 was approved by the Science and Technology Committee of Shihezi University, PR China and the Human Research Ethics Committee of Curtin University, Australia. Mothers who agreed to participate in the study signed the consent form and were informed of their rights to withdraw from the follow up process at anytime without prejudice. All the personal data collected were kept confidential.

All data analyses were carried out using the Statistical Package for Social Science (SPSS), advanced statistics, release 12.0 (SPSS for Windows, SPSS Inc., Chicago, IL, USA). Descriptive statistics and cross tabulations were generated for demographic factors and breastfeeding rates in 1994–1996. Life tables were used to analyse breastfeeding rates in 2003–2004. Chi-square tests were used to compare the breastfeeding rates in the two periods of time.

The definitions of breastfeeding used in this paper are [15-17]:

• 'Any breastfeeding': The child has received breastmilk (direct from the breast or expressed) with or without other drink, formula or other infant food;

• 'Exclusive breastfeeding': Breastfeeding while giving no other food or liquid, not even water, with the exception of drops or syrups consisting of vitamins, mineral supplements or medicine;

• 'Predominant breastfeeding': In addition to breastmilk the infant may receive small amounts of culturally valued supplement – water, water-based drinks, fruit juice and ritualistic fluids;

• 'Full breastfeeding' includes 'exclusive breastfeeding' and 'predominant breastfeeding'.

## Results

The demographic details of the study samples are shown in Table 1. The samples in the two periods of time were recruited from similar populations within the city of Shihezi. The first study was a cross sectional study and the response rate was 98%. The initial response rate for the second study was 97%. The proportion of mothers lost to follow-up at 1, 2, 3, 4 and 6 months were 3%, 6%, 10%, 13% and 20% respectively, that is 80% of mothers were followed to six months. This represents a response for 90.3% of the 'person-months' in the study.

**Table 1:** Demographic factors in 1994–1996 and 2003–2004, Shihezi, Xinjiang, PR China

		**1994–1996**		**2003–2004**	
**Characteristic**	**Values**	**n**	**%**	**n**	**%**

Ethnicity	Han Chinese	797	97	490	89.9
	Minorities	25	3	55	10.1

Maternal work	Farmer	138	16.8	60	10.9
	Housewife	68	8.3	174	32.0
	Worker	442	53.8	82	15.1
	Office Worker	118	14.3	82	15.1
	Others	56	6.8	142	26.8

Gestation	<37 Weeks	33	4	7	1.7
	37–41 Weeks	720	87.6	397	96.1
	42+ Weeks	69	8.4	9	2.2
	Missing			132	

Birth Order	1	737	89.7	483	88.8
	2+	85	10.3	61	11.2

Baby's Gender	Male	444	54	280	51.4
	Female	378	46	265	48.6

Birth Weight	<2500 g	36	4.4	11	2.1
	2500–2999 g	132	16.1	60	11.5
	3000–3499 g	358	43.5	191	36.5
	3500–3999 g	232	28.2	214	40.8
	4000 g+	64	7.8	48	9.2
	Missing			21	

Total		822		545	

The breastfeeding rates are detailed in Table 2. The 'any breastfeeding' rate at one month was 94% in 1994–1996 but was significantly lower (p < 0.01) at 86% in 2003–2004. After the first month, the 'any breastfeeding' rates in 1994–1996 were very similar to a decade later.

**Table 2:** Comparison of breastfeeding rates between 1994–1996 and 2003–2004 in Shihezi, PR China

	**1994–1996**			**2003–2004**		
**Baby's Age (months)**	**%**	**95% CI**	**n**	**%**	**95% CI**	**n**

**Full breastfeeding**						

1*	38.0	31.4, 44.6	208	57.3	52.6, 62.0	421
2**	36.0	27.6, 44.4	125	52.5	47.6, 57.4	407
3	32.5	26.0, 39.0	200	39.2	34.4, 44.0	392
4*	24.5	17.5, 31.5	147	9.7	6.7, 12.7	377
6*	9.2	4.4, 14.0	142	3.5	1.6, 5.4	348

**Any breastfeeding**						

1**	94.2	91.0, 97.4	208	85.6	82.2, 89.0	421
2	82.4	75.7, 89.1	125	84.3	80.8, 87.8	407
3	81.5	76.1, 86.9	200	82.8	79.1, 86.5	392
4	81.6	75.3, 87.9	147	81.9	78.0, 85.8	377
6	77.5	70.6, 84.4	142	76.2	71.7, 80.7	348

** p < 0.01, * p < 0.05 for differences between 1994–1996 and 2003–2004.

**Figure 2:**
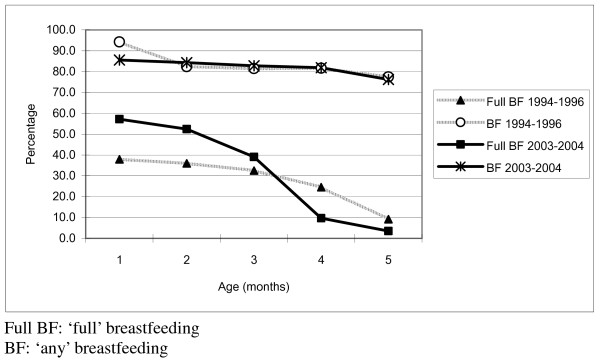
Comparison of breastfeeding rates (%) between 1994–1996 to 2003–2004, Shihezi, PR China.

The 'full breastfeeding' rate was significantly higher for the first two months in 2003–2004 than in 1994–1996, 57% compared to 38% at one month of age (p < 0.01). By four months of age the trend had reversed and the 'full breastfeeding' rate was lower in 2004 when compared to a decade earlier, as can be seen in Figure 2.

## Discussion

At four months the 'any breastfeeding' rate in both periods was the same (82%) and at six months in excess of three-quarters of women were continuing to breastfeed their infants. These rates are high compared to most developed countries but are lower than many countries at comparable income levels [8].

The increase in the 'full breastfeeding' rate in the first two months is an encouraging trend and may be the result of campaigns to promote 'exclusive breastfeeding' to four months. The fact that there were no increases in 'full breastfeeding' after four months, may reflect the emphasis of breastfeeding promotion campaigns in China during the 1990s and the impact of widespread advertising of infant formula. At this time the emphasis in China was on 'exclusive' or 'full breastfeeding' until 4 months, and not the six months that is now recommended by the World Health Organization [18].

In Xinjiang Province, the first Baby Friendly Hospital was accredited in 1995 in Urumqi, followed by one hospital in Shihezi in 1996. By 2001, all three hospitals in Shihezi had received accreditation as Baby Friendly Hospitals. As a result of the Baby Friendly Hospital Initiative, breastfeeding education courses were provided for nursing and medical staff and to all mothers. In 1996, all medical staff in the three Baby Friendly Hospitals participated in an 18 hour breastfeeding training course. Since that time, the staff has had three hours breastfeeding training every year to update their knowledge. All new staff has followed the same training process during their induction program. At the same time, mothers have been offered breastfeeding education programs during pregnancy and after the birth, including six hours of classroom attendance, three hours of video presentation and practical sessions postpartum.

In both of the studies the 'any breastfeeding' rates were slightly above 80% for the first four months. These rates are lower than in Karamay (a city in north Xinjiang) [19], Guangzhou [20]and Tibet [21], but similar to the rates in Wuhan [22]. Compared to Australia, the Shihezi 'any breastfeeding' rate was slightly higher [23,24], which in turn is higher than in Canada and the USA [25,26]. While the 'any breastfeeding' rates could usefully be maintained for longer, the more immediate problem seems to be the low 'full' or 'exclusive' breastfeeding rates.

However almost all mothers had introduced other foods and the rates of 'full breastfeeding' were low by four months. The 'full breastfeeding' rates at four months were 25% in 1994–1996 and 10% in 2003–2004, which were well below national and international targets [3]. The 'full breastfeeding' rates at four months were also lower than in Beijing and some other inland provinces [27]. With the implementation of breastfeeding promotion programs in the hospitals, it was hoped that the 'exclusive breastfeeding' rate and 'full' breastfeeding rate would be improved. However one reason for the lack of success of the breastfeeding promotion program may be that this period corresponded with an increase in prosperity in China. One consequence of the increase in purchasing power, even in a remote area such as Xinjiang, has been an increase in the advertising and availability of infant formula. These commercial activities provide a considerable challenge to breastfeeding promotion. Television advertisements of infant formula or children's food have a widespread influence on the population. For example, in Yili, the most remote county in the Shihezi area, it is now common practice for prospective parents to save their money and buy stocks of infant formula to prepare for their babies' births. The giving of infant formula as a gift at the infant's birth is becoming a common practice.

Traditional beliefs about breastfeeding also influence the duration of 'exclusive breastfeeding'. The majority of mothers believe that 'exclusive breastfeeding' cannot satisfy their baby's need for food until six months. Because of this the majority of babies were fed infant formula or solid food before four months [19]. Breastfeeding education during the antenatal period or in hospital does not seem to be enough to counter the dual influences of traditional beliefs and modern television commercials. Restricting advertising of formula and/or the use of television in health promotion programs may assist.

The main problem identified in these studies of breastfeeding in Shihezi was the early introduction of infant formula or other infant foods. Further studies are needed to identify detailed reasons associated with the short duration of 'exclusive' or 'full' breastfeeding.

Several limitations need to be considered when interpreting the results of this study. In the cohort study in 2003–2004, one group of babies were surveyed at approximately monthly intervals, while in the cross-sectional study in 1994–1996, different age groups were surveyed at one point of time. While the methods were different, similar questions were asked in both studies and some useful comparisons can be made. The results only apply to the Shihezi area and further studies are needed of other areas in the Xingjian Uygur Autonomous Region.

## Conclusion

The rate of breastfeeding at one month is significantly lower in 2003–2004 when compared to 1994–1996. The 'full breastfeeding' rates were initially higher, but after three months were then lower. The Chinese national breastfeeding targets were not reached in either period of the study. These studies show the need to further promote full or exclusive breastfeeding and further longitudinal studies are necessary to provide the detailed knowledge about risk factors required for health promotion programs.

## Competing interests

The author(s) declare that they have no competing interests.

## Authors' contributions

FX designed the research, collected and analyzed data, drafted the manuscript. XL designed the research and revised the manuscript. CX collected data, drafted the manuscript. CWB designed the research, drafted and revised the manuscript. JW collected data and drafted the manuscript. AHL analyzed data and revised the manuscript.
